# Rabeprazole‐amoxicillin dual therapy as first‐line treatment for *H pylori* eradication in special patients: A retrospective, real‐life study

**DOI:** 10.1111/hel.12717

**Published:** 2020-06-16

**Authors:** Wen Gao, Hui Ye, Xin Deng, Chi Wang, Ying Xu, Yixuan Li, Xuezhi Zhang, Hong Cheng

**Affiliations:** ^1^ GI Department Peking University First Hospital Beijing China; ^2^ TCM and Integrative Medicine Department Peking University First Hospital Beijing China

**Keywords:** amoxicillin, dual therapy, first‐line treatment, *Helicobacter pylori*, rabeprazole

## Abstract

**Background:**

The currently recommended quadruple regimens for *Helicobacter pylori* infection might not be appropriate for every patient, especially in elderly patients or those with multiple comorbidities.

**Objective:**

To evaluate the efficacy and safety of rabeprazole‐amoxicillin dual therapy in *H pylori*‐positive elderly patients or those with multiple comorbidities.

**Methods:**

From November 2013 to May 2017, the clinical data of *H pylori*‐positive patients ≥60 years old or with multiple comorbidities were collected and reviewed. All patients were given rabeprazole 10 mg three times a day and amoxicillin 1000 mg thrice a day (RA dual therapy) for 14 days as first‐line treatment. *H pylori* eradication was evaluated by ^13^C‐urea breath test 6 weeks after treatment. Adverse effects were recorded.

**Results:**

A total of 198 patients were enrolled, including 116 elderly patients and 82 patients with multiple comorbidities. Successful eradication was achieved in 90.9% (180/198, 95% CI: 86.1%‐94.2%) patients. Adverse effects, which were mainly mild (referring to skin rash, abdominal pain, and diarrhea), occurred in 22 patients (22/198, 11.1%) and resolved spontaneously.

**Conclusion:**

Dual therapy composed of rabeprazole and amoxicillin as a first‐line treatment appears to be effective and safe for *H pylori* infection in elderly patients or those with multiple comorbidities. Additional studies are needed to optimize the regimen.

## 1 INTRODUCTION

*Helicobacter pylori (H pylori)* infection is the primary cause of upper digestive diseases, typically gastritis, peptic ulcer, gastric adenocarcinoma, and mucosa‐associated lymphoid tissue lymphoma.^1^ Successful eradication has been widely proven to be beneficial for the recovery of gastric mucosa damage and a strategy for preventing gastric cancer.^2^, ^3^ However, *H pylori* eradication treatment still faces the global critical antibiotic resistance status despite decades of attempts. The prevalence of *H pylori* primary resistance revealed that resistance to clarithromycin, metronidazole, and levofloxacin was high and increased over time.^4^, ^5^ Sequential therapy and non‐bismuth concomitant therapy were thus compromised by antibiotic resistance and failed to fulfill the clinical requirements.^6^, ^7^, ^8^ Therefore, in most regions of China, 14‐d bismuth‐containing quadruple therapies have been considered the primary treatment regimens to treat *H pylori* infection under the circumstance of high antibiotic resistance, as recommended by the Fourth Chinese National Consensus Report on the management of *H pylori* infection.^9^ In the most recent Fifth Consensus Report, increasing the dosage of metronidazole to 1600 mg/d was suggested to enhance its clinical efficacy.^10^ However, higher doses of antibiotics lead to more adverse events (AEs), demand better tolerance, and therefore complicate the treatment decision, especially for those who are elderly or suffer from other systematic diseases with concomitant medications. A regimen with fewer medications is needed, especially for such patients.

Dual therapy was first designed to observe the interaction between proton‐pump inhibitors (PPI) and amoxicillin. The subsequent trials as first‐line therapy showed different treatment outcomes.^11^, ^12^ The dual regimens as salvage treatments acquired good results compared with those with bismuth quadruple therapy or triple therapies.^13^, ^14^ Effective gastric acid inhibition and sufficient amoxicillin were critical for the efficacy of dual therapy.^15^, ^16^ Amoxicillin works via a time‐dependent model; thus, frequent administration up to three or four times a day could achieve plasma concentrations above the MIC. Simultaneously, higher doses of the PPI could also offer a reliable pH (> 6 mostly) for treatment. Moreover, unlike clarithromycin, metronidazole, and levofloxacin, resistance to amoxicillin remains rare in the Asia‐Pacific region, including China.^17^ The PPI+ amoxicillin dual regimen might therefore be a good choice for *H pylori* treatment in China. A randomized controlled clinical trial conducted in China indicated that the eradication rate of dual therapy was similar to that of bismuth‐containing quadruple therapy, despite higher antibiotic resistance to clarithromycin rate in the dual therapy group.^18^ In our study, we aimed to evaluate the efficacy and safety of the dual therapy for *H pylori* eradication as a first‐line treatment for a group of special patients (defined as patients with advanced age or with multiple comorbidities) by retrospectively reviewing real clinical cases.

## 2 MATERIAL AND METHODS

### 2.1 Study design and participants

This was a retrospective, one‐arm study conducted at the Peking University First Hospital. From November 2013 to March 2017, we enrolled *H pylori*‐infected outpatients who could not tolerate triple or quadruple therapies because of (a) being elderly (≥60 years old) with or without comorbidities, (b) younger patients with comorbidities, such as nephritis, arrhythmia, cardiovascular disease taking statins, or warfarin competing with clarithromycin, and thus received rabeprazole‐amoxicillin (RA) dual therapy as the first‐line treatment. The exclusion criteria included patients receiving proton‐pump inhibitors (PPIs) or any antibiotics within 1 month before diagnosis. An upper gastrointestinal endoscopy was performed in all enrolled patients. The data on other factors such as age, sex, diagnosis of upper digestive disease, comorbidities, and concomitant medications were collected.

### 2.2 Diagnosis and eradication of *H pylori* infection and treatment regimen

*H pylori* infection was diagnosed as a positive ^13^C‐urea breath test (^13^C‐UBT), rapid urease test (RUT), or stool antigen test (SAT). The UBT was used as a common method for the detection of *H pylori* after eradication treatment and consensually recommended in China. It was also chosen as the method in follow‐up examination in our study performed at least 6 weeks after treatment.

RA dual therapy consisted of rabeprazole (10 mg) and amoxicillin (1000 mg) three times daily for 14 days. Rabeprazole was suggested to be taken half an hour before meals and amoxicillin postprandially.

### 2.3 Statistical analysis

Data collected were analyzed using IBM SPSS Statistics SPSS 20.0 software (IBM Corp.). Continuous variables were expressed as mean ± standard deviation, and categorical variables were expressed as numbers and percentages.

## 3 RESULTS

### 3.1 Background data of patients

A total of 198 patients receiving RA dual therapy as first‐line therapy were enrolled, including (a) 116 senior patients (age ≥ 60 years) of whom 106 patients had comorbidities, and (b) 82 younger patients all with comorbidities. The clinical parameters are shown in Table 1. The comorbidities were recorded in 188 cases, of which 105 patients had 1‐2 comorbidities, and 83 patients had ≥3 comorbidities concurrently. The main comorbidities (≥5 cases) are shown in Table 2.

**TABLE 1:** Baseline characteristics of patients

	Total (n = 198)	Elderly (n = 116)	Non‐elderly (n = 82)
Age, years	59.4 ± 13	68.6 ± 6	46.2 ± 10
Sex, male	70 (35.4)	43 (37.1)	27 (32.9)
Body mass index	22.8 ± 4	22.9 ± 4	22.7 ± 4
Smoking	26 (13.1)	18 (15.5)	8 (9.7)
Alcohol consumption	49 (24.7)	20 (17.2)	29 (35.4)
Immediate family history of malignancy			
Gastrointestinal cancer	24 (12.1)	14 (12.1)	10 (12.2)
Other malignancy	21 (10.6)	14 (12.1)	7 (8.5)
Endoscopy diagnosis			
Gastritis	173 (87.4)	98 (84.4)	75 (91.5)
Gastritis and gastric ulcer	10 (5.1)	8 (6.9)	2 (2.4)
Gastritis and duodenal ulcer	5 (2.5)	3 (2.5)	2 (2.4)
Gastritis and duodenal ulcer and esophagitis	1 (0.5)	1 (0.9)	0
Gastritis and duodenal ulcer and duodenitis	1 (0.5)	1 (0.9)	0
Gastric ulcer	2 (1.0)	1 (0.9)	1 (1.2)
Gastric ulcer and esophagitis	1 (0.5)	1 (0.9)	0
Duodenal ulcer and duodenitis	1 (0.5)	1 (0.9)	0
Complex ulcer	4 (2.0)	2 (1.7)	2 (2.4)

**TABLE 2:** Major Comorbidities (cases ≥ 5)

Comorbidity	n
Hypertension	73
Dyslipidemia	35
Diabetes mellitus	27
Hepatitis B virus infection	20
Coronary heart disease	18
Cancer	14
Nodular goiter	12
Gallstone	12
Arrhythmia	10
Hyperthyroidism	9
Atherosclerosis	9
Urticaria	7
Hashimoto's thyroiditis	6
Gallbladder polyps	6
Osteoporosis	6
Nephritis	6
Fatty liver	6
Anemia	5
Asthma	5
Kidney stone	5

In total, 132 patients concomitantly and continually took medications, such as statins, hypotensors, hypoglycemics, antiplatelet drugs, antiviral drugs, immunosuppressors, and antitumor drugs. Sixty‐seven patients took 1‐2 types of medications, 51 patients took 3‐4 types of medications, and 14 patients took ≥5 types of medications.

### 3.2 Eradication rates of *H pylori* infection

**FIGURE 1:**
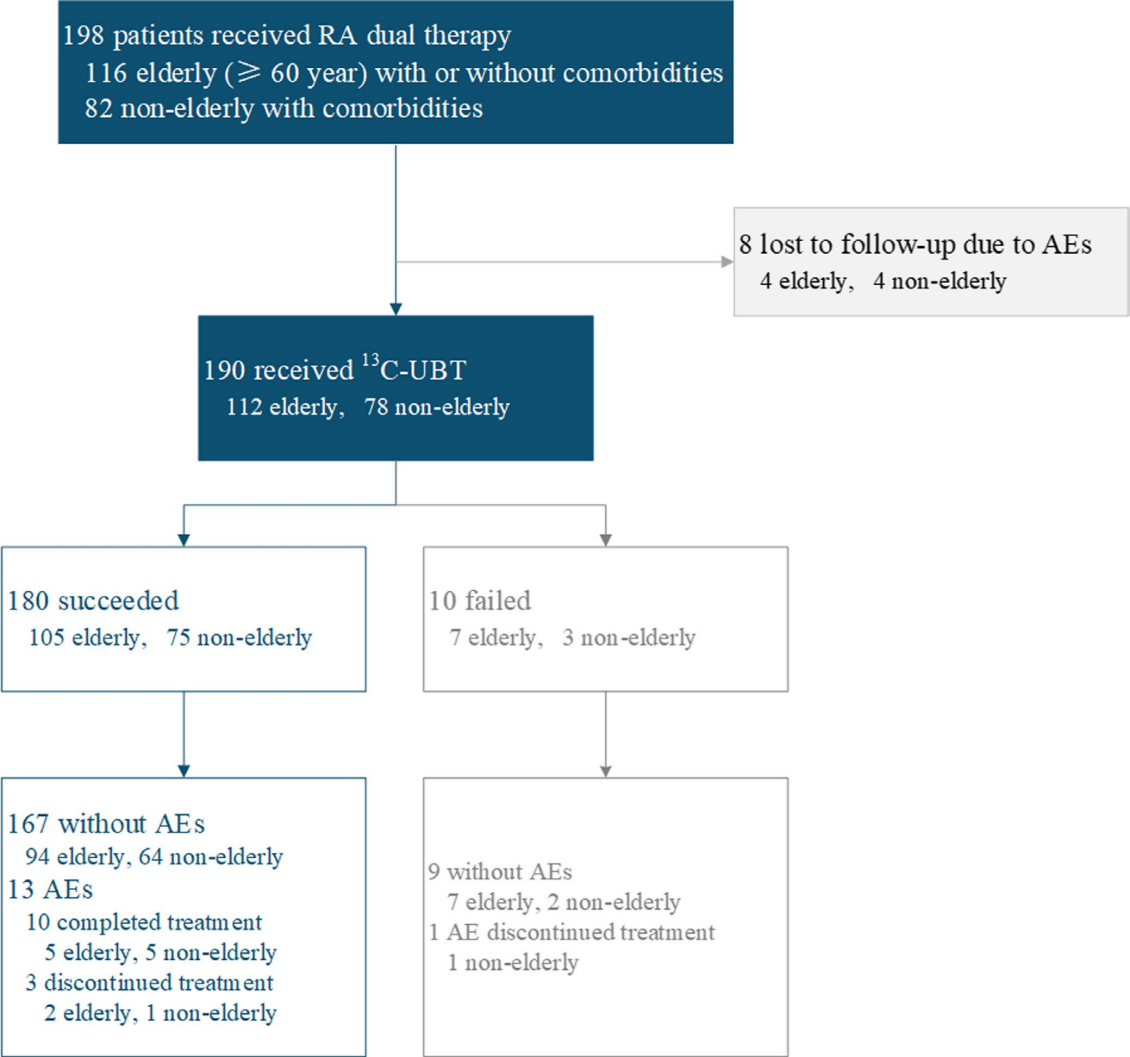
Flowchart of the study. In total, 198 subjects were reviewed. A total of 180 cases successfully eradicated *H pylori*, in which 167 patients were free of any AEs. The RA dual therapy failed in 10 patients. Eight patients were lost to follow‐up

In 198 patients enrolled, 190 patients received the assessment of eradication using ^13^C‐UBT, and 8 patients were lost to follow‐up. A total of 180 patients achieved successful eradication. The eradication rates were 90.9% (180/198, 95% CI 86.1%‐94.2%) in all cases (flowchart in Figure 1). Ten patients failed to the treatment, of whom 3 had diabetes mellitus, 2 had autoimmune diseases, and 2 had chronic kidney diseases.

In the subgroup of 116 elderly patients, the RA dual therapy succeeded in 105 cases and failed in 7 cases, while 4 patients were lost to follow‐up. The eradication rate was 90.5% (105/116, 95% CI 83.8%–94.6%) in 116 elderly patients. In the subgroup of 82 patients aged <60 years with comorbidities, treatment succeeded in 75 cases and failed in 3 cases. Four patients were lost to follow‐up. The eradication rate was 91.5% (75/82, 95% CI 83.4%‐95.8%).

### 3.3 AEs

Twenty‐two patients (22/198, 11.1%), 11 in the elderly patient group and 11 in the non‐elderly patient group, experienced AEs. Nine of these 22 patients had ≥3 comorbidities. The most common AEs were rash, abdominal pain, and diarrhea (Table 3). All AEs completely disappeared without any intervention after treatment.

**TABLE 3:** Cases of AEs

Manifestation of AEs	n
Rash	9
Abdominal pain	5
Diarrhea	5
Headache	3
Dizziness	2
Fatigue	2
Nausea	2
Leukopenia	1
Fever	1
Palpitation	1

Ten of 22 patients with AEs completed the whole treatment. The remaining 12 patients discontinued treatment, in whom 3 patients achieved successful eradication despite incomplete treatment with a duration of 7‐12 days, 1 patient failed, and 8 patients were lost to follow‐up (details in Table 4). Four of 12 patients who did not complete the treatment had chronic kidney diseases, and 2 patients had chronic liver diseases.

**TABLE 4:** Details of AEs

Gender	Age (year)	AEs manifestation	Occurrence date in treatment	Treatment continuation (Y/N)	Successful eradication (Y/N)
Female	38	Rash	8th	Y	Y
Male	33	Rash	3rd	Y	Y
Female	58	Leukopenia	5th	Y	Y
Female	67	Abdominal pain	7th	Y	Y
Female	69	Fever, headache	2nd	Y	Y
Female	59	Rash	4th	Y	Y
Male	61	Rash	4th	Y	Y
Female	63	Dizziness	3rd	Y	Y
Female	21	Abdominal pain, diarrhea	4th	Y	Y
Female	61	Diarrhea	2nd	Y	Y
Female	34	Rash, abdominal pain, diarrhea	13th	N	Y
Female	60	Rash	8th	N	Y
Male	74	Nausea	7th	N	Y
Female	49	Headache, nausea	5th	N	N
Female	69	Rash	3rd	N	—
Female	30	Rash	9th	N	—
Male	69	Palpitation	8th	N	—
Female	43	Dizziness, fatigue	3rd	N	—
Female	70	Abdominal pain	10th	N	—
Female	38	Rash, headache, fatigue, diarrhea	8th	N	—
Female	71	Diarrhea	8th	N	—
Female	30	Abdominal pain,	3rd	N	—

Note

Abbreviations: —: lost to follow‐up;N, no; Y, yes.

## 4 DISCUSSION

The antibiotic resistance of *H pylori* has increased in the past decades, which is the main cause of treatment failure. It has been reported that only 16.3% of isolated *H pylori* strains are susceptible to all tested antibiotics, while dual or multiple resistances are commonly observed with metronidazole, clarithromycin, and fluoroquinolones.^19^ The primary and secondary antibiotic resistance rates of amoxicillin are stabilized at a low level (1%) in the Western Pacific region.^20^ Elderly patients fail their intended eradication therapy because of previous multiple exposures to antibiotics. Another multi‐region prospective study concluded that compared to patients <40 years old, elderly patients exhibit higher resistance rates to clarithromycin, azithromycin, levofloxacin, and moxifloxacin. The resistance rate to amoxicillin remained low in both groups.^21^ Higher antibiotic resistance in the elderly population reduced the efficacy of therapies containing these antibiotics.

Bismuth‐containing quadruple therapy could partially overcome antibiotic resistance. However, AEs occurred in more than 50% of the levofloxacin‐containing bismuth quadruple therapy and standard bismuth‐containing quadruple therapy, compared with 26.2% in triple therapy.^22^ There were also many competing conditions when it came to the elderly or people with multiple diseases compared to younger ones, considering the possibility of interaction with concomitant medicines.^23^, ^24^ Moreover, renal dysfunction and chronic liver disease might affect drug metabolism and induce drug accumulation. The presence of arrhythmia, prolonged QTc interval, and heart diseases might also limit the use of clarithromycin because of a potentially increased risk of heart problems or death. Clarithromycin inhibits cytochrome P450 enzyme 3A4 (CYP3A4), which was associated with a doubled risk of hospitalization with rhabdomyolysis or other statin‐related AEs according to a systematic review recently.^25^ Calcium channel blockers (CCBs), which are used in patients with hypertension, were also metabolized by CYP3A4. Clarithromycin might interact with CCBs and be associated with a higher risk of acute renal injury.^26^ Fluoroquinolones might cause a significant decrease in blood glucose, resulting in hypoglycemic coma, particularly in elderly people and patients with diabetes. In our study, hypertension, dyslipidemia, and diabetes mellitus were the top three comorbidities, and the choice of antibiotics was, thus, restricted. As for patients older or with multiple comorbidities, a dual regimen with fewer antibiotics would be an option.

Dual therapy, which was composed of PPI+ amoxicillin, was not traditional therapy, even though it had already been used as early as 1998.^27^ Amoxicillin was proven to be sensitive in most cases^4^ and could work especially in a relatively high pH environment.^28^ The dose of amoxicillin was assumed as ideal in 3 g/d in most reports.^27^ The other factor that affected the efficacy of treatment was the ability to reliably maintain a relatively high intragastric pH, where the potency of PPIs was involved. Rabeprazole was introduced to the dual regimen because it was less likely to interact with other drugs, such as clopidogrel, warfarin, and tacrolimus, due to its cytochrome P450 2C19 (CYP2C19) polymorphism. CYP2C19 and IL‐1β polymorphisms are not significant independent factors of *H pylori* eradication using rabeprazole‐based hybrid therapy.^29^ Rabeprazole has also been reported to possess potential antibacterial properties in an in vitro study.^30^ As standard PPI therapy (twice‐daily dosing) often failed to induce a reliable high intragastric pH, rabeprazole was designed to be given three times per day in our study, which was 30 mg daily, to enhance the acid inhibition effect.^31^

At present, the efficacy of the dual regimen remains controversial in different studies, probably due to different durations, dosages, and dose intervals of PPI and amoxicillin.^27^ It was supposed that 14 days of dual therapy (40 mg omeprazole and 1000 mg amoxicillin three times daily) was more effective than 10 days of therapy (40 mg esomeprazole and 1000 mg amoxicillin three times daily).^32^ The trials conducted in Shanghai and Taiwan demonstrated that dual regimens with a double dose of PPI or amoxicillin could achieve a higher eradication rate.^33^, ^34^, ^35^, ^36^ A 14‐day high‐dose dual therapy consisting of a standard or double dose of PPI and a total of 3 g/d amoxicillin was recommended as a salvage regimen for *H pylori* treatment in the ACG Clinical Guideline.^37^ Given the ground of similar efficacy,^38^ less pill exposure, lower risk of major drug interactions, and lower potential for AEs, amoxicillin‐based dual therapy may be considered as an alternative therapy in special patients.^39^ In our study, a 14‐day dual regimen consisting of 10 mg rabeprazole and 1000 mg amoxicillin 3 times per day achieved a satisfactory eradication rate of 90.9% as the first‐line treatment.

In the case of the 10 patients who failed the eradication treatment, 1 patient had a history of cigarette smoking, 1 patient discontinued the treatment on the 5th day due to AEs, and 6 patients took other medications concurrently. Diabetes mellitus was the most common comorbidity observed in these 10 patients. Previous studies indicated that age ≥ 45 years, insufficient compliance, a short duration of treatment, diabetes mellitus, and cigarette smoking were independent predictive factors of treatment failure.^40^, ^41^, ^42^

AEs occurred in 11.1% (22/198) of patients involved in dual therapy, which remained mild and tolerable similar to most reports.^27^ Skin rash, diarrhea, and abdominal pain were the most frequently occurred AEs. All AEs disappeared spontaneously after treatment. 13 patients (13/22) with AEs had successful eradication, and 3 of them discontinued the treatment between the 7th and 12th day of treatment. One case showed failure of treatment, and 8 cases were lost to follow‐up.

There are limitations to our study. First, a prospective, well‐designed study would always be better than a retrospective study. At the beginning of our study, the RA regimen was considered an alternative regimen for patients with old age or multiple comorbidities. However, the continuous involvement of more and more patients since the eradication rate is encouraging. A prospective study has been designed and performed recently. Second, it was performed in one hospital as a single‐center study. Third, there was no control group for comparison. Fourth, the patients involved in the study were special, either elderly or with multiple comorbidities. A randomized multicenter trial would be designed to observe its effect in a future study. Since most of the dual therapy studies published were performed in Asia, there might be bias caused by population characteristics.

In conclusion, the RA dual regimen was effective and safe as a first‐line treatment for *H pylori* infection in special patients. Our study revealed the real clinical practice situation and actual demand of a group of special patients who were older or had multiple comorbidities. A 14‐day RA dual regimen was an ideal choice, which was effective and safe regardless of antibiotic resistance background or CYP2C19 polymorphism status. To improve the efficacy of dual therapy, further studies are warranted, which examine the following effects: (a) to increase the dose of PPI to enhance acid inhibition to induce a more reliable intragastric pH environment^31^; (b) to change PPI to vonoprazan, which might be more powerful in combination with amoxicillin in some reports^43^; and (c) to give amoxicillin more frequently since it is time‐dependent, such as 0.75 g four times per day.

## CONFLICT OF INTEREST

All authors declare no conflicts of interest related to this article.

## AUTHOR CONTRIBUTION

Wen Gao and Hui Ye contributed equally to manuscript rewriting and data analysis; Xin Deng, Chi Wang, Ying Xu, Yixuan Li, and Xuezhi Zhang assisted in collecting patient data; Hong Cheng contributed to designing the study, providing clinical data, and reviewing the drafts and final versions of the manuscript.

## References

[1] Malfertheiner P , Megraud F , O'Morain CA , et al. Management of *Helicobacter pylori* infection‐the Maastricht V/Florence Consensus Report. Gut. 2017;66(1):6‐30. 27707777 10.1136/gutjnl-2016-312288

[2] Jin X , Li YM . Systematic review and meta‐analysis from Chinese literature: the association between *Helicobacter pylori* eradication and improvement of functional dyspepsia. Helicobacter. 2007;12(5):541‐546. 17760723 10.1111/j.1523-5378.2007.00520.x

[3] Wang J , Xu L , Shi R , et al. Gastric atrophy and intestinal metaplasia before and after *Helicobacter pylori* eradication: a meta‐analysis. Digestion. 2011;83(4):253‐260. 21282951 10.1159/000280318

[4] Gao W , Cheng H , Hu F , et al. The evolution of *Helicobacter pylori* antibiotics resistance over 10 years in Beijing. China. Helicobacter. 2010;15(5):460‐466. 21083752 10.1111/j.1523-5378.2010.00788.x

[5] Hu Y , Zhu Y , Lu NH . Primary Antibiotic Resistance of *Helicobacter pylori* in China. Dig Dis Sci. 2017;62(5):1146‐1154. 28315035 10.1007/s10620-017-4536-8

[6] Qian J , Ye F , Zhang J , et al. Levofloxacin‐containing triple and sequential therapy or standard sequential therapy as the first line treatment for *Helicobacter pylori* eradication in China. Helicobacter. 2012;17(6):478‐485. 23067317 10.1111/j.1523-5378.2012.00993.x

[7] Zhou L , Zhang J , Chen M , et al. A comparative study of sequential therapy and standard triple therapy for *Helicobacter pylori* infection: a randomized multicenter trial. Am J Gastroenterol. 2014;109(4):535‐541. 24642580 10.1038/ajg.2014.26

[8] Hong J , Shu X , Liu D , et al. Antibiotic resistance and CYP2C19 polymorphisms affect the efficacy of concomitant therapies for *Helicobacter pylori* infection: an open‐label, randomized, single‐centre clinical trial. J Antimicrob Chemother. 2016;71(8):2280‐2285. 27107097 10.1093/jac/dkw118

[9] Liu WZ , Xie Y , Cheng H , et al. Fourth Chinese National Consensus Report on the management of *Helicobacter pylori* infection. J Dig Dis. 2013;14(5):211‐221. 23302262 10.1111/1751-2980.12034

[10] Liu WZ , Xie Y , Lu H , et al. Fifth Chinese National Consensus Report on the management of Helicobacter pylori infection. Helicobacter. 2018;23(2):e12475. 29512258 10.1111/hel.12475

[11] Kashifard M , Malekzadeh R , Siavoshi F , et al. Continuous and more effective duodenal ulcer healing under therapy with bismuth and two antibiotics than with dual therapy comprising omeprazole and amoxicillin. Eur J Gastroenterol Hepatol. 1998;10(10):847‐850. 9831406 10.1097/00042737-199810000-00006

[12] Labenz J , Gyenes E , Ruhl GH , Borsch G . Amoxicillin plus omeprazole versus triple therapy for eradication of *Helicobacter pylori* in duodenal ulcer disease: a prospective, randomized, and controlled study. Gut. 1993;34(9):1167‐1170. 8406147 10.1136/gut.34.9.1167 PMC1375447

[13] Miehlke S , Hansky K , Schneider‐brachert W , et al. Randomized trial of rifabutin‐based triple therapy and high‐dose dual therapy for rescue treatment of *Helicobacter pylori* resistant to both metronidazole and clarithromycin. Aliment Pharmacol Ther. 2006;24(2):395‐403. 16842467 10.1111/j.1365-2036.2006.02993.x

[14] Yang JC , Lin CJ , Wang HL , et al. High‐dose dual therapy is superior to standard frst‐line or rescue therapy for *Helicobacter pylori* infection. Clin Gastroenterol Hepatol. 2015;13:895‐905. 25460556 10.1016/j.cgh.2014.10.036 PMC4404168

[15] Labenz J , Gyenes E , Rühl GH , et al. Omeprazole plus amoxicillin: efficacy of various treatment regimens to eradicate *Helicobacter pylori* . Am J Gastroenterol. 1993;88(4):491‐495. 8470626

[16] Bayerdörffer E , Miehlke S , Mannes GA , et al. Double‐blind trial of omeprazole and amoxicillin to cure *Helicobacter pylori* infection in patients with duodenal ulcers. Gastroenterology. 1995;108(5):1412‐1417. 7729633 10.1016/0016-5085(95)90689-4

[17] Kuo Y‐T , Liou J‐M , El‐Omar EM , et al. Primary antibiotic resistance in *Helicobacter pylori* in the Asia‐Pacific region: a systematic review and meta‐analysis. Lancet Gastroenterol Hepatol. 2017;2(10):707‐715. 28781119 10.1016/S2468-1253(17)30219-4

[18] Yang J , Zhang YI , Fan L , et al. Eradication efficacy of modified dual therapy compared with bismuth‐containing quadruple therapy as a first‐line treatment of *Helicobacter pylori* . Am J Gastroenterol. 2019;114(3):437‐445. 30807294 10.14309/ajg.0000000000000132

[19] Song Z , Zhang J , He L , et al. Prospective multi‐region study on primary antibiotic resistance of *Helicobacter pylori* strains isolated from Chinese patients. Dig Liver Dis. 2014;46(12):1077‐1081. 25220697 10.1016/j.dld.2014.08.038

[20] Savoldi A , Carrara E , Graham DY , et al. Prevalence of antibiotic resistance in *Helicobacter pylori*: a systematic review and meta‐analysis in World Health Organization regions. Gastroenterology. 2018;155(5):1372‐1382.e17. 29990487 10.1053/j.gastro.2018.07.007 PMC6905086

[21] Liu D‐S , Wang Y‐H , Zeng Z‐R , et al. Primary antibiotic resistance of *Helicobacter pylori* in Chinese patients: a multiregion prospective 7‐year study. Clin Microbiol Infect. 2018;24(7):780.e5‐780.e8. 10.1016/j.cmi.2017.11.010 29138101

[22] Kahramanoğlu Aksoy E , Pirinçci Sapmaz F , Göktaş Z , et al. Comparison of *Helicobacter pylori* eradication rates of 2‐week levofloxacin‐containing triple therapy, levofloxacin‐containing bismuth quadruple therapy, and standard bismuth quadruple therapy as a first‐line regimen. Med Princ Pract. 2018;26(6):523‐539. 10.1159/000484930 PMC5848476 29131124

[23] Norgard NB , Mathews KD , Wall GC . Drug‐drug interaction between clopidogrel and the proton pump inhibitors. Ann Pharmacother. 2009;43:1266‐1274. 19470853 10.1345/aph.1M051

[24] Cizginer S , Ordulu Z , Kadayifci A . Approach to Helicobacter pylori infection in geriatric population. World J Gastrointest Pharmacol Ther. 2014;5(3):139‐147. 25133042 10.4292/wjgpt.v5.i3.139 PMC4133439

[25] Hougaard Christensen MM , Bruun Haastrup M , Øhlenschlæger T , et al. Interaction potential between clarithromycin and individual statins—a systematic review. Basic Clin Pharmacol Toxicol. 2020;126(4):307‐317. 31628882 10.1111/bcpt.13343

[26] Gandhi S , Fleet JL , Bailey DG , et al. Calcium‐channel blocker‐clarithromycin drug interactions and acute kidney injury. JAMA. 2013;310(23):2544‐2553. 24346990 10.1001/jama.2013.282426

[27] Gao CP , Zhang D , Zhang T , et al. PPI‐amoxicillin dual therapy for *Helicobacter pylori* infection: an update based on a systematic review and meta‐analysis. Helicobacter. 2020;e12692 10.1111/hel.12692 32314468

[28] Casciaro M , Navarra M , Inferrera G , et al. PPI adverse drugs reactions: a retrospective study. ClinMol Allergy. 2019;17:1. 10.1186/s12948-019-0104-4 PMC6337765 30675130

[29] Lin TJ , Lee HC , Lin CL , et al. CYP2C19 polymorphism has no influence on rabeprazole‐based hybrid therapy for *Helicobacter pylori* eradication. World J Clin Cases. 2018;6(12):514‐520. 30397607 10.12998/wjcc.v6.i12.514 PMC6212610

[30] Sharara AI . Rabeprazole: the role of proton pump inhibitors in *Helicobacter pylori* eradication. Expert Rev Anti Infect Ther. 2005;3(6):863‐870. 16307499 10.1586/14787210.3.6.863

[31] Graham DY , Lu H , Dore MP . Relative potency of proton‐pump inhibitors, *Helicobacter pylori* therapy cure rates, and meaning of double‐dose PPI. Helicobacter. 2019;24(1):e12554. 30440097 10.1111/hel.12554 PMC6905108

[32] Zullo A , Ridola L , Francesco VD , et al. High‐dose esomeprazole and amoxicillin dual therapy for first‐line *Helicobacter pylori* eradication: a proof of concept study. Ann Gastroenterol. 2015;28(4):448‐451. 26423014 PMC4585390

[33] Ren L , Lu H , Li HY , et al. New dual therapy for primary treatment of *Helicobacter pylori* infection: a prospective randomized study in Shanghai, China. J Dig Dis. 2014;15(11):622‐627. 25205201 10.1111/1751-2980.12186

[34] Yang JC , Wang HL , Chern HD , et al. Role of omeprazole dosage and cytochrome P450 2C19 genotype in patients receiving omeprazole‐amoxicillin dual therapy for *Helicobacter pylori* eradication. Pharmacotherapy. 2011;31(3):227‐238. 21361732 10.1592/phco.31.3.227

[35] Yu L , Luo L , Long X , et al. High‐dose PPI‐amoxicillin dual therapy with or without bismuth for first‐line *Helicobacter pylori* therapy: a randomized trial. Helicobacter. 2019; 24(4):e12596. 31111580 10.1111/hel.12596

[36] Tai W‐C , Liang C‐M , Kuo C‐M , et al. A 14 day esomeprazole‐ and amoxicillin‐containing high‐dose dual therapy regimen achieves a high eradication rate as first‐line anti‐*Helicobacter pylori* treatment in Taiwan: a prospective randomized trial. J Antimicrob Chemother. 2019;74(6):1718‐1724. 30768161 10.1093/jac/dkz046

[37] Chey WD , Leontiadis GI , Howden CW , et al. ACG clinical guideline: treatment of *Helicobacter pylori* infection. Am J Gastroenterol. 2017;112(2):212‐239. 28071659 10.1038/ajg.2016.563

[38] Zhang YI , Zhu Y‐J , Zhao Z , et al. Efficacy of modified esomeprazole‐amoxicillin dual therapies for Helicobacter pylori infection: an open‐label, randomized trial. Eur J Gastroenterol Hepatol. 2020;32(5):563‐568. 31851093 10.1097/MEG.0000000000001646

[39] Nguyen CT , Davis KA , Nisly SA , et al. Treatment of *Helicobacter pylori* in special patient populations. Pharmacotherapy. 2019;39(10):1012‐1022. 31400244 10.1002/phar.2318

[40] Perri F , Villani MR , Festa V , et al. Predictors of failure of *Helicobacter pylori* eradication with the standard 'Maastricht triple therapy'. Aliment Pharmacol Ther. 2001;15(7):1023‐1029. 11421878 10.1046/j.1365-2036.2001.01006.x

[41] Labenz J , Leverkus F , Börsch G . Omeprazole plus amoxicillin for cure of *Helicobacter pylori* infection. Factors influencing the treatment success. Scand J Gastroenterol. 1994;29(12):1070‐1075. 7886394 10.3109/00365529409094890

[42] Yao CC , Kuo CM , Hsu CN , et al. First‐line Helicobacter pylori eradication rates are significantly lower in patients with than those without type 2 diabetes mellitus. Infect Drug Resist. 2019;12:1425‐1431. 31239721 10.2147/IDR.S194584 PMC6554512

[43] Furuta T , Yamade M , Kagami T , et al. Dual therapy with vonoprazan and amoxicillin is as effective as triple therapy with vonoprazan, amoxicillin and clarithromycin for eradication of *Helicobacter pylori* . Digestion. 2019;1‐9. 10.1159/000502287 31434101

